# DOTA: Deep Learning Optimal Transport Approach to Advance Drug Repositioning for Alzheimer’s Disease

**DOI:** 10.3390/biom12020196

**Published:** 2022-01-24

**Authors:** Jacqueline Chyr, Haoran Gong, Xiaobo Zhou

**Affiliations:** 1 Center for Computational Systems Medicine, School of Biomedical Informatics, University of Texas Health Science Center, Houston, TX 77030, USA; jacqueline.chyr@uth.tmc.edu; 2 West China Biomedical Big Data Center, West China Hospital, Sichuan University, Chengdu 610041, China; gonghaoran@wchscu.cn

**Keywords:** Alzheimer’s disease, drug repositioning, deep learning, multi-modal autoencoder, optimal transport problem, reactome, diseasome, circadian patterns

## Abstract

Alzheimer’s disease (AD) is the leading cause of age-related dementia, affecting over 5 million people in the United States and incurring a substantial global healthcare cost. Unfortunately, current treatments are only palliative and do not cure AD. There is an urgent need to develop novel anti-AD therapies; however, drug discovery is a time-consuming, expensive, and high-risk process. Drug repositioning, on the other hand, is an attractive approach to identify drugs for AD treatment. Thus, we developed a novel deep learning method called DOTA (Drug repositioning approach using Optimal Transport for Alzheimer’s disease) to repurpose effective FDA-approved drugs for AD. Specifically, DOTA consists of two major autoencoders: (1) a multi-modal autoencoder to integrate heterogeneous drug information and (2) a Wasserstein variational autoencoder to identify effective AD drugs. Using our approach, we predict that antipsychotic drugs with circadian effects, such as quetiapine, aripiprazole, risperidone, suvorexant, brexpiprazole, olanzapine, and trazadone, will have efficacious effects in AD patients. These drugs target important brain receptors involved in memory, learning, and cognition, including serotonin 5-HT2A, dopamine D2, and orexin receptors. In summary, DOTA repositions promising drugs that target important biological pathways and are predicted to improve patient cognition, circadian rhythms, and AD pathogenesis.

## 1. Introduction

Alzheimer’s disease (AD) is a degenerative disease characterized by memory loss, cognitive function decline, functional impairment, and other neuropsychological symptoms. It is the leading cause of age-related dementia, affecting over 35 million people worldwide. It is one of the costliest chronic diseases, with a global healthcare cost of $305 billion as estimated by the World Alzheimer’s Association [1,2]. The prevalence and cost of AD continues to rise as our population ages.

Currently, there are only five FDA-approved drugs for AD treatment. They include four acetylcholinesterase inhibitors and one N-methyl-D-aspartate receptor antagonist, memantine [3]. These drugs are prescribed to improve memory, attention, reason, language, and the ability to perform simple tasks by affecting neurochemicals involved in carrying messages between brain nerve cells. Unfortunately, these treatments are only palliative because they do not slow down or halt the disease progression [4,5,6], and none can cure AD [7]. Therefore, there is an urgent need to identify novel anti-AD therapies.

Drug discovery is a time-consuming, laborious, expensive, and high-risk process. It usually takes 10 to 15 years to develop a new drug, with a 2.01% average success rate of developing a new molecular entity [7,8,9]. The cost of drug development is increasing every year. There is a trend of overinterpretation of earlier phase clinical trials and preclinical data which led to failed large-scale trials, such as tarenflurbil [10,11], dimebon [12], and semegestat [13]. Additionally, there is a lack of targeted recruitment for trial participants, resulting in a vastly heterogeneous cohort of patients with various comorbidities which confounds the data and leads to unclear efficacies. Drug repositioning, on the other hand, is an attractive approach for discovering drugs for diseases. The repositioning of existing approved clinical drugs has accelerated novel drug discoveries for many diseases [14]. Various data-driven computational or analyzing methods were reported for drug repurposing, however, these attempts at drug repurposing for AD resulted in failure [15,16,17,18,19,20]. Previous AD drug repositioning failures may be due to a lack of sufficient target engagement, unrealized toxic effects, and incomplete understanding of the complexity of AD pathogenesis [21,22]. Comprehensive multi-modal computational approaches are needed to reposition effective FDA-approved drugs for AD.

Most diseases, including AD, are a result of complex interactions between multiple genetic and/or environmental factors. The increasing availability of bioinformatics data and high-throughput interaction screening has pioneered a new science called “network medicine”, which focuses on the interrelationships of genes, proteins, and external environment. Incorporating multiple data sources can boost the accuracies of in silico drug repositioning; however, it is a challenge to capture complex and non-linear structures. To address this challenge, we employed multimodal auto-encoders (MAE) in our approach [23,24,25]. MAE preserves the non-linear network structures by applying multiple layers of non-linear functions. It is effective in denoising systems and constructing useful representation from sparse data. It is also scalable because it can learn low-dimensional drug features from all networks in a fully unsupervised way, independently of drug-disease prediction tasks.

Additionally, disturbances of the circadian rhythms have long been associated with many neurological and psychiatric diseases, including Alzheimer’s disease (AD), Parkinson’s disease (PD), and Huntington’s disease (HD) [26]. Roughly 80% of AD patients over 65 years old suffer from circadian rhythm disorders, such as disturbances in thermoregulation and sleep-wake cycles [27,28]. AD patients also exhibit disturbances in the timing and duration of the sleep cycle, primarily manifested as increased wakefulness at night and increased sleep during the day, which can progress to a loss of day–night variation [29]. Sundowning, manifested as agitation or delirium in the evening, is a common symptom in AD patients, especially in the mid-disease stage [30,31]. In advanced stages, the severe disruption or reversal of normal sleep cycles constitutes the primary cause for institutionalization [32]. Other studies have showed that degeneration of circadian activity patterns and/or sleep fragmentation occur in the early, pre-symptomatic phase during AD pathogenesis, and displayed predictive values for later development of cognitive deficits, pathological Aβ deposition, and dementias [33,34]. An epidemiologic study of daily activity of over 1200 initially cognitively-normal older women demonstrated that diminished circadian rhythm amplitude, robustness, or phase delay were associated with increased risk of developing dementia during the 5-year follow-up period [33].

Accumulating evidence supports a possible causal relationship between disruption of circadian rhythms and AD [26,32,35,36]. Recent experiments demonstrated that sleep deprivation caused a striking increase in the Aβ plaque burden in mice that express AD-associated mutant forms of human amyloid precursor protein (APP) and develop Aβ plaques with age [37,38]. A subsequent study found an increase in cortical Aβ plaque burden following chronic sleep deprivation in mice that express human APP, PS1, and human Tau transgenes [39]. New findings in the fruit fly and rodent models indicate that the deletion of the clock genes *Bmal1, Clock, Per1,* and *Cry1* may cause an accelerated aging phenotype characterized by an earlier decline in cognitive functions [40,41,42]. Mouse genetic and pharmacological studies reveal causal roles of clock manipulation in AD pathology and neurodegeneration. In mammals, a group of core clock components, including positive (CLOCK, BMAL1, RORs) and negative (PERs, CRYs, REV-ERBs) factors, formed interlocked transcription/translation feedback loops [43], and the intact circadian oscillator is required for neuronal maintenance and cognitive functions [44]. Thus, FDA-approved drugs with circadian effects may be efficacious for AD treatment.

Here, we present DOTA: a novel and robust network-based deep learning approach to reposition drugs for AD treatment. DOTA considers drug targets, side effects, and associations with other diseases in its predictions, which increases the discovery of mechanistically effective drugs for AD. Through seamless integration of multiple drug networks and implementation of advanced algorithms, DOTA identifies several promising drug candidates for AD treatment. A closer analysis found that our drug predictions improved circadian patterns, agitated behaviors, psychosis, and even delayed cognition-decline in AD patients. Our tool can also be broadly applied to investigate drug candidates for other diseases and have a boundless clinical impact.

## 2. Materials and Methods

### 2.1. Assembling Drug–Target–Sideeffects–Disease Networks

Heterogeneous networks were assembled from multiple clinically or experimentally validated drug databases. Drug–drug interactions were collected from DrugBank databases. There were 1519 unique drugs with 290,836 drug–drug interactions. Drug–gene/protein interactions were collected from DrugBank [45], the Therapeutic Target Database [46], and the PharmGKB databases [47]. Only experimentally validated binding affinities (inhibition potency, dissociation constant, median effective concentration, and median inhibitory concentration ≤ 10 µM) from ChEMBL [48], BindingDB [49], and IUPHAR/BPS Guide to PHARMACOLOGY databases [50,51] were included. Proteins that cannot be mapped to a unique UniProt accession number were excluded. Drug–side-effects and adverse drug events were collected from clinically reported information from MetaADEDB [52], CTD [53], SIDER [54], and OFFSIDES [55] databases. There were 382,041 drug–side-effects associations for the 1519 unique drugs. Drug–AD interactions were extracted from DrugBank [56] and repoDB [57] databases. Drug names (chemical, generic, or commercial) were standardized by Medical Subject Headings (MeSH) [58] and Unified Medical Language System (UMLS) [59], and converted to DrugBank ID.

In addition to Drug–Drug, Drug–Gene/Protein, Drug–Side-Effects, and Drug–AD interactions described above, five additional drug networks were assembled. They include: (1) similarities in drug chemical structures, (2) similarities in side-effects, (3) similarities in protein sequence of drug targets, (4) similarities in biological functions, and (5) similarities in therapeutic and clinical properties. Similarities in drug chemical structures and similarities in drug’s side effects of drug pairs were computed using the Tanimoto coefficient T, which is widely used in drug discovery and development [60]. Molecular fingerprints (166-bit 2D structures) of the 1519 drugs were computed using Open Babel [61]. If two drugs have a and b fragment bits, with c fragment bits found in both drugs, then the similarity of these two drugs is defined as T=c/(a+b−c). Likewise, if two drugs have a and b side effects, with c side effects associated with both drugs, then the similarity of side-effects of two drugs is calculated by the same equation. The Tanimoto coefficient ranges from 0 to 1, where 0 represents no similarities and 1 represents high similarities. Similarities in drug targets (proteins) were calculated by averaging the similarities of all target protein sequences of a drug pair. Canonical protein sequences of drug targets were obtained from UniProt database [62]. Protein sequence similarities for drug pairs were calculated using the Smith–Waterman algorithm [63], which performs local sequence alignment by comparing protein segments of all possible lengths. Similarities in biological functions were computed using a graph-based semantic similarity measure algorithm called GOSemSim [64,65]. Experimentally validated evidence (semantic annotations) of biological processes, molecular functions, and cellular components were obtained from Gene Ontology (GO) [65]. The overall similarities of two drugs, A and B, (in terms of biological functions of the drugs’ target genes) is calculated by averaging all pairs of drug target-coding genes a and b with a∈A and b∈B. Similarities in therapeutic and clinical effects of drug pairs were calculated with similarities in Anatomical Therapeutic Chemical (ATC) classification systems codes [66,67].

### 2.2. Network Representation and Fusion

Incorporating multiple networks of different data types can offer great insights for drug repositioning; however, integrating highly heterogeneous and non-linear data is a challenge. For homogenous networks (i.e., drug–drug interaction network and five drug-drug similarity networks described in Section 2.1), random walk-based network representation [68] was applied to mitigate the sparsity of individual network types and to capture each network’s structural information:

(1) $$p_{k}=\omega \times p_{k-1}M+\left(1-\omega \right)p_{0}$$

where M is the transition matrix that captures the transition probabilities between vertices, ω is the probability that the random walk procedure will continue, and $p_{k}$ is a row vector after a walk-length k. That is, the vertices of a network are first ordered randomly, and then the relationship between vertices of a graph is expressed in a linear sequence. Vertices are uniformly sampled by first selecting one vertex, $v_{1}$, as the current vertex, then randomly selecting the new vertex, $v_{2}$, from all the neighbors of the current vertex, $v_{1}$. Next, the newly selected vertex, $v_{2}$, is set as the current vertex and this vertex sampling process repeats until the number of vertices within one sequence reaches a pre-set walk-length k. The random walk procedure will continue with a probability of ω, and will return to the original vertex and restart the procedure with a probability 1−ω. By repeating the random walk process of each node in the network and summing the recurrence relation of each random walk, we obtain a probabilistic co-occurrence matrix C based on the sampled linear sequences. Then, the co-occurrence matrixes C are factorized, and the associations are represented as positive pointwise mutual information (PPMI) matrixes, where $N_{r}$ is the number of rows and $N_{c}$ is the number of columns:

(2) $$\mathrm{PPMI}=\max\left(\log\frac{C_{\left(i,j\right)}\times \sum_{i}^{N_{r}}\sum_{i}^{N_{c}}C_{\left(i,j\right)}}{\sum_{i}^{N_{r}}C_{\left(i,j\right)}\times \sum_{i}^{N_{c}}C_{\left(i,j\right)}},\;0\right)$$

For heterogeneous networks (i.e., drug–gene/protein, drug–side-effects, and drug–AD networks), the Jaccard similarity coefficient [60] was calculated first before attaining the PPMI matrixes. Jaccard similarity is commonly used for characterizing the similarities between two sets of samples, A and B.

(3) $$J\left(A,B\right)=\frac{\left|A\cap B\right|}{\left|A\cup B\right|}$$

The resulting PPMI matrices are then fused together using Multimodal Auto-Encoder (MAE). MAE is a special type of neural network that is composed of an encoder where input data is transformed into low-dimensional features, and a decoder where those features are mapped back to the input data. Here, MAE was used to integrate the different drug networks into a compact, low-dimensional feature representation common to all networks. We followed the formulation for MAE as previously described in deepNF: deep network fusion [69].

### 2.3. Drug–Disease Predictions with Optimal Trasport

To infer new drug–AD associations, DOTA uses a variational autoencoder (VAE). Drug features extracted from the embedding layer of MAE and known drug–disease interactions are encoded and decoded by a generator and discriminator network. The autoencoder may have a denoised understanding of the drug features. Since the drug feature is numerical, the loss function is the mean squared error:

(4) $$MSE={\left(D-\hat{D}\right)}^{2}/2$$

where D is the original drug features and $\hat{D}$ represents the reconstructed drug features. We fine-tuned the variational autoencoder to unravel the latent relation between drugs and diseases. The input is a row vector representing a specific disease and the columns in the vector represent the possibility that a drug can treat the disease.

Optimal transport theory derives from the allocation of resources problem. The goal is to allocate resources from one distribution to another distribution. It represents the relation between two distributions and can be used to compute loss in machine learning. The optimal transport problem can be defined as follows:

(5) $$L=argmin_{\gamma }\iint_{x,y}\gamma \left(x,y\right)c\left(x,y\right)dxdy$$

(6) $$\int_{x}\gamma \left(x,y\right)dx=P\left(x\right)$$

(7) $$\int_{y}\gamma \left(x,y\right)dy=Q\left(y\right)$$

where P(x) and Q(y) are two distributions, γ(x,y)=γ(x|y)p(x)=γ(y|x)q(y) is the joint distribution, and c(x,y) is a pre-defined distance. Wasserstein loss is used to measure the difference between the input and output in this step since the input and output are considered a distribution:

(8) $$\mathrm{Wasserstein}\;\mathrm{loss}=\sum_{i=0,j=0}\gamma \left(x_{i},{\hat{x}}_{j}\right)\left|x_{i},{\hat{x}}_{j}\right|\;$$

Specifically, $\gamma \left(x_{i},{\hat{x}}_{j}\right)$ is the transition cost in optimal transport theory, and $\left|x_{i},{\hat{x}}_{j}\right|$ is the distance between input and output. We use geometry distance to calculate the distance:

(9) $$\mathrm{Geometry}\;\mathrm{distance}\;={\left(x_{i}-\hat{x_{i}}\right)}^{2}$$

The final loss function consists of three parts. The first part is the Wasserstein loss, which calculates the distance (Equation (8)). The second part is a regularization, which is used to constrain the intermediate results of the VAE. Finally, the third part is the auxiliary, which is used to help determine potential AD drugs.

(10) $$\mathrm{regularization}=\sum 1+2\log\left(\sigma \right)-\sigma^{2}-\mu^{2}$$

(11) $$\mathrm{auxiliary}=\sum x_{i}\log\left({\hat{x}}_{j}\right)+(1-x_{i})\log\left(1-{\hat{x}}_{j}\right)$$

(12) Final loss=Wasserstein loss+α×regularization+0.1×auxiliary

Our approach preserves the non-linear network structure through the application of multiple layers of non-linear functions and predicts potential drug–disease associations using informative, fused drug features and known (clinically reported or FDA-approved) drug–disease associations.

### 2.4. Analysis of the Human Reactome

The drug targets (protein targets mapped to their corresponding genes) of the top 20 predicted drugs were extracted from DrugBank [45], the Therapeutic Target Database [46], and the PharmGKB databases [47]. Then, their biological functions and signaling pathways were analyzed using Reactome [70,71]. Reactome is a collection of known biological processes and pathways. The human reactome consists of 10,720 proteins, 13,804 complexes, 13,890 reactions, and 2546 pathways. It is a manually curated and peer-reviewed pathway database, visualization, and interpretation resource.

### 2.5. Analysis of the Human Diseasome

The human disease network was obtained from Goh, K. et al. [72]. Briefly, the diseasome consists of all known genetic disorders and all known disease genes in the human genomes. Diseases and genes are then connected by a link if mutations in the gene are implicated in the disease. In the human disease network, each node represents a disease, and two diseases are connected to each other if they share at least one gene which mutations are associated with both diseases. The human diseasome is visualized with Gephi v0.9.2, a network visualization and exploration software [73].

## 3. Results

### 3.1. Overview of DOTA

**Figure 1:**
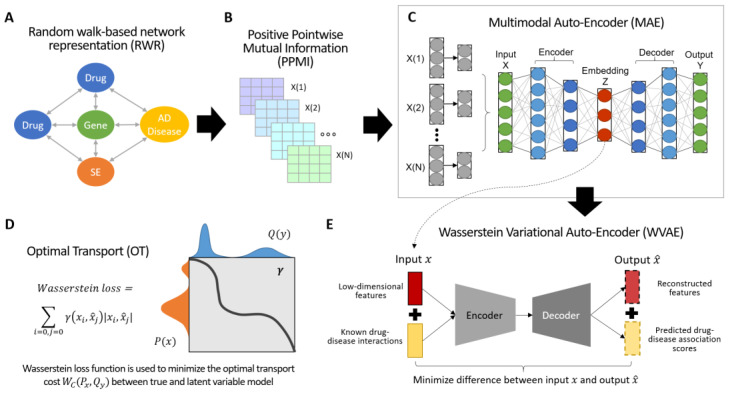
Overview of DOTA. (**A**) Drug networks (drug–drug, drug–gene, drug–side-effects, drug–disease, and five other drug–drug similarities) are first converted into high-quality vector representation with a random walk-based procedure. (**B**) Next, the associations of the factorized co-occurrence matrixes are represented as PPMI matrixes. (**C**) The PPMI matrixes are then fused together into a low-dimensional feature representation using an unsupervised multimodal auto-encoder. (**D**) The optimal transport problem used in the second autoencoder part of DOTA. A Wasserstein loss function is used to minimize the optimal transport cost $W_{C}\left(P_{X},\;P_{G}\right)$ between the input and output. (**E**) The drug information from the embedding layer of MAE is extracted and used as drug features to predict new drug-disease associations. A WVAE is used to encode and decode the drug-associations.

To identify drugs with the potential to be efficacious in treating patients with AD, a novel computational approach called DOTA was developed. DOTA is a network-based, deep learning approach that integrates multimodal networks, captures the complex and highly non-linear networks structures, and systematically infers potential associations between FDA-approved drugs and AD. The pipeline of DOTA is shown in Figure 1. This approach consists of network representation step, and two major autoencoders: (1) Multi-modal Auto-Encoder (MAE) to first fuse multiple drug networks together, and (2) Wasserstein Auto-Encoder (WAE) to optimally transport the extracted low-dimensional information from the embedding layer of the MAE into reconstructed features and predicted drug–AD association scores. The goal is to identify and reposition drugs currently used for other conditions, as well as drugs from failed clinical trials, for AD treatment.

### 3.2. Constructing and Integrating Drug Networks

Heterogeneous drug networks were assembled from multiple clinically or experimentally validated drug databases, including drug–drug interactions, drug–gene/protein interactions, drug–side-effects, and drug–disease interactions. In addition, five more drug networks were included: (1) similarities in drug chemical structures, (2) similarities in side-effects, (3) similarities in protein sequence of drug targets, (4) similarities in biological functions, and (5) similarities in therapeutic and clinical properties.

For homogenous networks (i.e., drug–drug interaction network and five drug–drug similarity networks), random walk-based network representation was applied to mitigate the sparsity of individual network types and to capture each network’s structural information. For heterogeneous networks (i.e., drug–gene/protein, drug–side-effects, and drug–AD networks), the Jaccard similarity coefficient was calculated. Next, the co-occurrence matrixes are factorized, and the associations are represented as positive pointwise mutual information (PPMI) matrixes. The multiple PPMI matrices are then fused together using a Multimodal Auto-Encoder (MAE). This resulted in a compact, low-dimensional feature representation common to all networks.

### 3.3. Drug Predictions and Association Using Optimal Transport

The second autoencoder in DOTA is a variational autoencoder (VAE) that uses the elegant geometric properties of the optimal transport problem and the Wasserstein distances to predict drug–disease associations between FDA-approved drugs and AD. This approach minimizes the Wasserstein distance between the distributions of encoded information from the embedding layers of the multimodal autoencoder step and the reconstructed output. The VAE uses drug information from the embedding layer of the MAE to predict new drug–disease associations.

### 3.4. Repositionig Results and Validation

**Figure 2:**
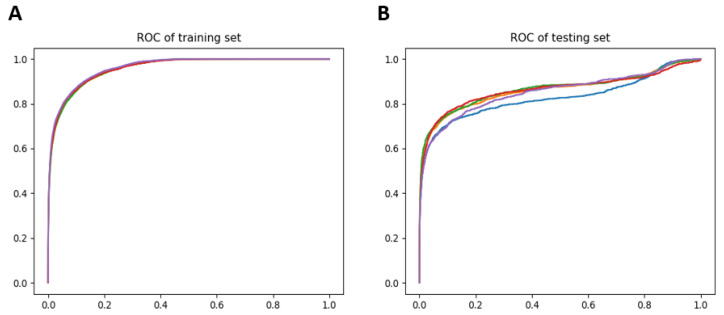
Performance of DOTA. Drug–disease combinations were partitioned into training (**A**) and testing sets (**B**) at an 80:20 ratio. Five-fold cross validation was performed and the individual ROC for each of the five folds are shown for both training and testing sets.

To evaluate the accuracy and reliability of our method, DOTA was applied on known drug–disease interactions for all diseases. In total, there are 1519 drug–disease samples and they are allocated into training and testing sets in an 80:20 ratio. A five-fold cross validation was performed. The average area under the receiver operating characteristic curve (AUROC) for the training and testing datasets are 0.95 and 0.85, respectively. The receiver operating characteristic curve (ROC) for the training and testing sets are shown in Figure 2.

**Figure 3:**
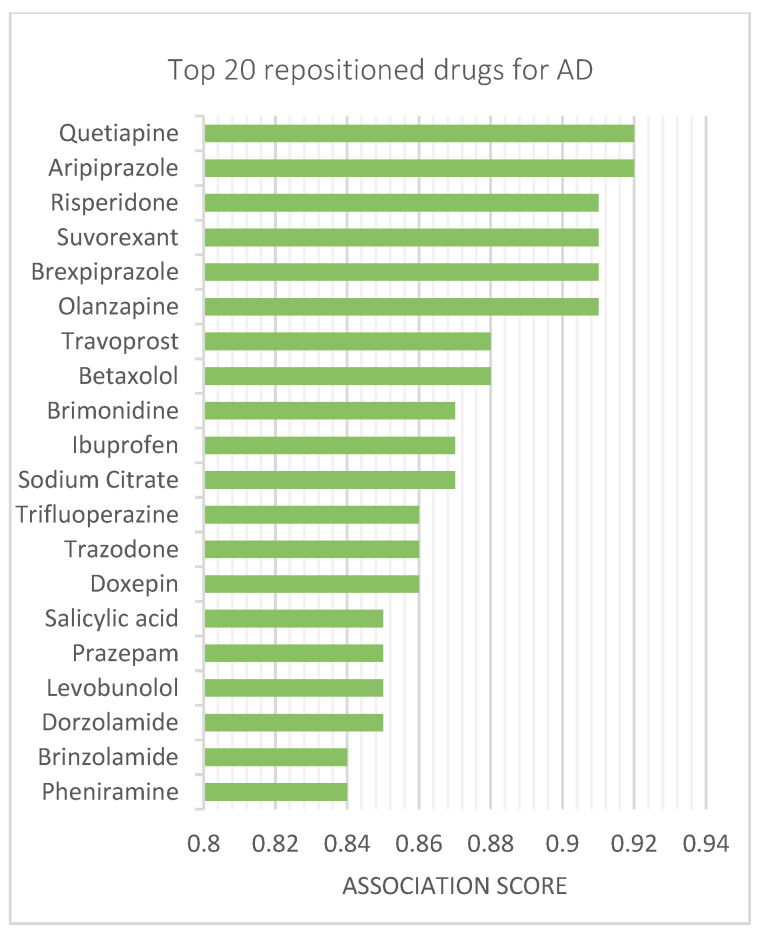
Top drug–disease associations for AD. Top 20 drug candidates for AD, as predicted by DOTA, ranked by association scores.

The top ten DOTA-predicted drug candidates for AD include: aripiprazole, quetiapine, risperidone, suvorexant, brexpiprazole, olanzapine, travoprost, betaxolol, brimonidine, and ibuprofen. The top repositioned drugs and their association scores are shown in Figure 3. The association scores for all drugs are provided in Supplementary Table S1. Risperidone, aripiprazole, and quetiapine, which are atypical antipsychotics for the treatment of schizophrenia and bipolar disorder, were predicted to have a potential effect on AD by both DOTA and another deep learning repositioning tool called deepDR [74]. Unlike deepDR, which uses a cross-entropy function, DOTA uses a Wasserstein loss function. Additionally, DOTA included a drop-out layer to avoid overfitting.

### 3.5. Reactome Analysis—Functional and Biological Targets of Repositioned Drugs

**Figure 4:**
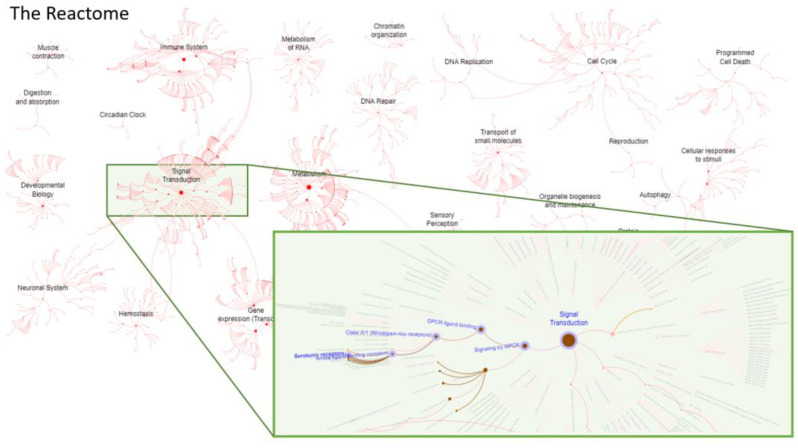
The human reactome of drug targets. The drug targets of the top 20 predicted drug candidates for AD treatment was analyzed and visualized with Reactome Pathway Browser. In the insert, the serotonin receptor pathway is shown as an example. The darker the edges are, the more enriched the top gene targets are.

To evaluate the biological functions and biochemical impact of candidate repositioned drugs, the drug targets and their corresponding pathways were analyzed. Using the Reactome database, which is a collection of signaling and metabolic molecules and their relationships, we identified several important biological pathways and processes that are affected by the top predicted drugs [70,71]. The drug targets of antipsychotic drugs, such as quetiapine, aripiprazole, risperidone, suvorexant, brexpiprazole, and trazadone, were involved in signal transduction pathways, such as serotonin receptor, adrenoceptors, dopamine receptors, and histamine receptor signaling pathways (Figure 4). In total, the 62 drug targets were involved in 191 signaling pathways. The full list of the pathways is provided in Supplementary Table S2.

### 3.6. Quantifying Anticholinergic Burden and Sedative Load of Repositioned Drugs

The anticholinergic burden and sedative load of the top 20 candidate AD drugs are examined. Anticholinergic burden is defined as the accumulation of one or more anticholinergic medication with increased risk of medication-related adverse side effects. Sedative load is defined as medication-related effects of sleepiness, lethargy, drowsiness, and reduced psychomotor processing. Using data from the AntiCholinergic and Sedative Burden Catalog (ACSBC) [75], the anticholinergic burden and sedative load of the top DOTA-predicted drugs are quantified in older adults. In Table 1, candidate AD drugs are categorized into high, moderate, low, or no anticholinergic and sedative activity based on currently available information. Ten of the top 20 candidate drugs have anticholinergic or sedative effects.

### 3.7. Diseasome Analysis—Relationships between AD and Other Diseases

**Figure 5:**
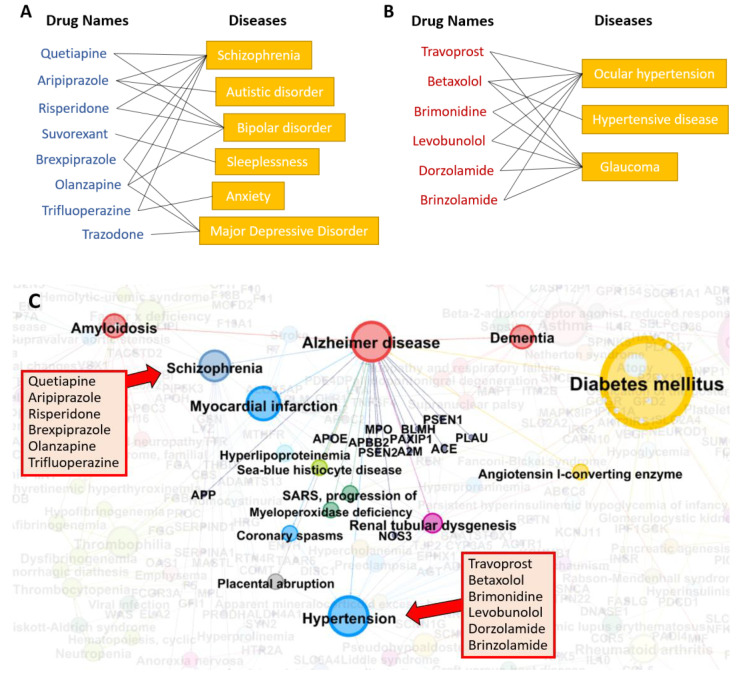
Known drug–disease associations. Among the top 20 predicted drugs, eight drugs are known to treat cognitive diseases, such as schizophrenia and bipolar disorder (**A**), and six drugs are known to treat hypertensive disorders (**B**). (**C**) The diseasome is shown for AD. Colored nodes are disorders that share risk factors or mechanisms with AD and faded nodes are other disorders that are not related to AD. The size of the nodes represents the number of edges connecting to the disorder in the diseasome.

A diseasome is a network of diseases linked by known disease–gene associations. Genes associated with similar disorders are more likely to have physical interactions between their products and have higher expression profiling similarity for their transcripts. We evaluated the relationship between AD and other diseases by analyzing the human diseasome. As suspected, there are connections between AD and dementia, amyloidosis, and schizophrenia as shown in Figure 5C. There are also relationships between AD and heart diseases such as myocardial infarction and hypertension. DOTA predicted several candidate AD drugs that are known to treat schizophrenia, including quetiapine, aripiprazole, risperidone, brexpiprazole, olanzapine, and trifluoperazine (Figure 5A). Other predicted drugs, such as travoprost, betaxolol, brimonidine, levobunolol, dorzolamide, and brinzolamide, are known to treat ocular hypertension and hypertensive disease (Figure 5B). This analysis suggests that DOTA’s predicted drugs are efficacious for AD treatment due to their success in treating related diseases that share similar risk factors and mechanisms.

### 3.8. Clinical Analysis of Candidate AD Drugs and Their Effects on Circadian Patterns

Clinical analysis revealed that several candidate drugs have a circadian effect. The top three DOTA-predicted drugs (i.e., Risperidone, Aripiprazole, and Quetiapine) was also predicted by others to be effective in AD [74]. Risperidone selectively antagonizes serotonin (5-HT) effects via cortical 5-HT2 receptor, and, to a lesser extent, competes with dopamine at the limbic dopamine D2 receptor. It is found to be effective for wandering and disturbed sleep/wake patterns in AD [91]. Risperidone is also found to reset the circadian rhythm in mice, which may be extended to clinical studies to adjust the circadian rhythm in mental disorders [92]. Aripiprazole regulates dopamine activity by reducing it when it is high and increasing it in areas where it is low, which helps with symptoms such as hallucination and poor motivation, respectively. A low dose of aripiprazole was found to correct the circadian rhythm, and reduced nocturnal sleep time in patients with delayed sleep phase syndrome [93]. One study has also found an improvement of patient’s circadian rhythm sleep disorders along with the stabilization of the patient’s bipolar disease with aripiprazole treatment [94]. In addition, this drug activates BMAL1, an important clock gene, and causes a shortening effect on the period of circadian rhythm [95,96]. Quetiapine is often used to treat psychosis in elderly patients with AD. It was found to increase sleep duration and efficiency, delay final wake time, and reduce within-day variability [97]. These DOTA-predicted drugs have beneficial clinical impact in AD patients and may be effective therapies for AD treatment.

## 4. Discussion

There is a tremendous need for the identification of effective therapies for AD treatment. Thus, we developed a novel deep learning approach, called DOTA, to reposition FDA approved drugs for AD treatment. Unlike any other drug repositioning methods, DOTA uses optimal transport to calculate the distance between the input and output while minimizing the cost. Our approach identified promising antipsychotic and hypertensive drugs for AD treatment, such as quetiapine, aripiprazole, risperidone, betaxolol, and brimonidine, to name a few. Several predicted drugs are expected to be beneficial for AD patients due to their pharmacological mechanisms of action. For example, suvorexant is a dual antagonist of orexin receptors OX1R and OX2R, and sleep deprivation and sleep-promoting orexin signaling were found to influence the levels of AD-related proteins, Aβ and tau, in interstitial fluid or cerebrospinal fluid, respectively, during the sleep/wake cycle [38,98,99,100,101].

Both DOTA and a different drug repositioning approach called deepDR predicted three overlapping drug candidates for AD treatment [74]. They include three atypical antipsychotics: risperidone, aripiprazole, and quetiapine. These drug candidates are commonly used in the treatment of schizophrenia and bipolar disorder, which are disorders that are closely related to AD and share gene mutations and risk factors. Surprisingly, all three drugs were also found to have circadian effects in patients. Since disruption in circadian rhythm is common among AD patients and there is evidence supporting a causal relationship [26,32,35,36], treating AD patients with drugs that also have an effect on circadian rhythmicity may improve cognition, sleep, and AD pathogenesis.

Interestingly, one drug candidate that was identified with DOTA, but not with deepDR, is trazadone. This drug is often used to treat depression and insomnia, and it functions as a serotonin receptor antagonist. In a recent clinical study, AD patients showed stabilization of circadian rhythms and exhibited a significant improvement in relative rhythm amplitude after two weeks of trazadone treatment [102]. Trazadone was also found to have a positive effect on dementia and delayed cognitive decline in 25 AD patients [103]. This may be due to its effect on augmenting slow-wave sleep and its target on serotonin and norepinephrine, which are both known to be dysfunctional in AD [104,105]. Another analysis revealed that there may be a dose-independent dual effect of trazadone on human cognition, with acute utilization leading to impaired cognition while long-term use preventing cognition deterioration [106]. Drugs with anticholinergic or sedative properties are commonly prescribed to patients with polypharmacy [75,107]. While these medications are needed to treat co-occurring chronic diseases, some studies have noted that long-term exposure to anticholinergic and sedative medication may contribute to cognitive and physical decline [108,109]. These experimental and clinical results support the accuracy of DOTA in predicting potentially effective drugs for AD treatment; however additional studies are needed to evaluate the safety and long-term effects of these drugs on human cognition.

Despite our vigilant efforts, there are several limitations to our work. First, it is difficult to evaluate the performance of our model because our purpose is to identify novel drug–disease associations. In other words, negative pairs, i.e., drug–disease pairs with no known associations, may have unrealized associations and should not be treated as negative samples in our model performance evaluations. Secondly, drug networks, such as drug targets and drug side effects, may be incomplete due to the ever-growing discoveries made experimentally and in clinical trials. Currently, there is a still a lack of preclinical information for several predicted drugs in appropriate models. As more information become available, our model can be re-trained to offer more accurate and appropriate drug predictions for AD. Third, the likelihood of success is still dependent on several factors such as real-world heterogeneity of clinical conditions and patient backgrounds, including other underlying conditions and medication.

In summary, DOTA identified FDA-approved drugs that are predicted to be effective for AD treatment. These drugs target several important signaling pathways related to AD, including serotonin and dopamine signaling pathways. Our discoveries would not be possible without the development of a robust and powerful deep learning approach that uses optimal transport in the prediction of new drug–disease associations from comprehensive drug features and known drug–disease associations.

## 5. Conclusions

The emergence of high-throughput molecular technologies, combined with exponential growth in the amount of biomedical data, has created unprecedented opportunities to expand our understanding of drug functions and drug–target interactions, leading to the development of a novel deep learning Drug repositioning approach using Optimal Transport for Alzheimer’s disease called DOTA and the subsequent identification of several drug repositioning candidates for AD treatment.

## Figures and Tables

**Table 1:** Anticholinergic and sedative burden of candidate drugs. Top repositioned drugs with anticholinergic burden or sedative load are shown. Drugs are categorized as high, moderate, low, and no anticholinergic burden and sedative load based on the ACSBC cumulative scale. References of scales and metrics used in the categorization are included.

Drug Name	Anticholinergic Burden	Sedative Load
Quetiapine	Moderate [76]	Moderate [77]
Aripiprazole	Low [78,79,80]	Moderate [81]
Risperidone	Low [76,78,79,80,82,83,84,85,86,87]	Moderate [77]
Suvorexant	No	High [77]
Olanzapine	Moderate [76,82]	Moderate [77]
Travoprost	No [79]	No
Betaxolol	Low [76,79,88]	Low [77]
Ibuprofen	No [79,80,89]	Low [77]
Trifluoperazine	High [78,82]	High [77]
Trazodone	Low [76,78,79,80,82,84,86,87]	Moderate [77]
Doxepin	High [78,79,83,89,90]	Moderate [81]

## Data Availability

The data used for model training and testing, as well as the source codes for DOTA, are provided at https://github.com/fawer/DOTA. Last accessed 26 December 2021.

## Supplementary Materials

The following are available online at https://www.mdpi.com/article/10.3390/biom12020196/s1: Table S1, S1-DOTA-association-scores.csv; Table S2, S2-reactome-pathways.csv.
